# Secular trends in cryoglobulinemia mortality in the USA in the era of direct-acting antivirals

**DOI:** 10.1186/s13075-022-02720-1

**Published:** 2022-02-12

**Authors:** Qianyu Guo, Jinfang Gao, Jiaoniu Duan, Ruihong Hou, Tsung-Hsueh Lu, Liyun Zhang

**Affiliations:** 1 Department of Rheumatology, Third Hospital of Shanxi Medical University, Shanxi Bethune Hospital, Shanxi Academy of Medical Sciences, Tongji Shanxi Hospital, Taiyuan, 030032 Shanxi China; 2 Department of Public Health, College of Medicine, National Cheng Kung University, Tainan, Taiwan

**Keywords:** Cryoglobulinemia, Direct-acting antivirals, Hepatitis C virus, Mortality trends

## Abstract

**Background:**

Hepatitis C virus (HCV) is the main etiology of cryoglobulinemia with mortality around 25%. Little is known on the changes in cryoglobulinemia mortality after the introduction of direct-acting antivirals (DAA) for treatment of HCV in 2014 in the USA.

**Methods:**

We used the multiple-cause mortality files compiled by the National Center for Health Statistics to calculate cryoglobulinemia mortality from 1999 to 2018. The proportionate mortality ratio (PMR) of cryoglobulinemia cases with HCV and those with autoimmune diseases was computed to assess the impact of introduction of DAA.

**Results:**

We identified 1299 people aged ≥ 20 years who died with cryoglobulinemia between 1999 and 2018. The cryoglobulinemia mortality (deaths per million) declined from 1999 (0.4) to 2010 (0.22) and mildly increased to 2014 (0.26), and then decreased abruptly from 2014 to 2018 (0.19) with annual percent change of − 14.3%. The proportion of cryoglobulinemia patients with HCV was 39% (118/302) in 2009–2013 and 26% (81/310) in 2014–2018, with a PMR of 0.67 (95% CI 0.50–0.89). By contrast, the proportion of cryoglobulinemia patients with systemic autoimmune diseases was 2.6% (8/302) in 2009–2013 and 4.2% (13/310) in 2014–2018, with a PMR of 1.58 (95% CI 0.66–3.82).

**Conclusion:**

The changes in cryoglobulinemia mortality during the past two decades are mainly related to the aging and dying of the “baby boomer” cohort who had a high HCV prevalence and to the introduction of a DAA in 2014.

## Introduction

Cryoglobulins are immunoglobulins that precipitate in vitro at temperatures below 37 °C, and cryoglobulinemia is defined as the persistent presence of cryoglobulins in serum. Hepatitis C virus (HCV) is the main etiology accounting for 80–90% of cryoglobulinemia cases [1]. Although a review of a cryoglobulinemia case series conducted in 2008, it indicated heterogeneous mortality results from 16% to 66% [2]. Later prognosis studies of patients with cryoglobulinemia and HCV suggested more consistent mortality results (ranging from 21 to 29%) relating to life-threatening organ damage involving renal failure, pulmonary hemorrhage, gastrointestinal ischemia, and cerebral ischemia or hemorrhage [3–7].

Several novel direct-acting antivirals (DAAs) for treatment of HCV were approved by the US Food and Drug Administration in late 2013, and many clinical trials have indicated a good response from patients with HCV and cryoglobulinemia [8–10]. However, changes in the epidemiology of cryoglobulinemia before and after the introduction of DAAs have not been extensively examined in the USA. Therefore, this study was conducted to examine secular trends in cryoglobulinemia mortality within the USA from 1999 to 2018, with a particular focus on changes occurring after the introduction of DAAs in 2014.

## Methods

### Data sources and case definition

We used the multiple-cause mortality files compiled by the National Center for Health Statistics (NCHS) to identify decedents aged 20 years and above who died between 1999 and 2018 and whose death certificates mentioned cryoglobulinemia [11]. The multiple-cause mortality files are available in two data formats: entity axis and record axis codes [11]. We used record axis codes in this study because they are edited for NCHS core multi-cause tabulations.

The *International Classification of Diseases, Tenth Revision* (*ICD-10*) was adopted by the USA in 1999, and the *ICD-10* codes associated with relevant diseases are as follows: D891 for cryoglobulinemia, B171 or B182 for HCV, M30–M36 for systemic autoimmune disease, M32 for systemic lupus erythematosus (SLE), and M350 for Sjogren’s syndrome (SS).

### Mortality rates

The cryoglobulinemia mortality rate was calculated by counting cases in which cryoglobulinemia was recorded either as the underlying or contributory cause of death on the death certificate as the numerator and general population as denominator. We calculated the age-standardized mortality rate using the age structure of the USA for the year 2000 as the standard population. The proportionate mortality rates (%) for HCV, SLE, and SS were also computed, i.e., number of death with mention both HCV and cryoglobulinemia divided by number of death with mention cryoglobulinemia (similarly, SLE + cryoglobulinemia/cryoglobulinemia and SS + cryoglobulinemia/cryoglobulinemia).

### Statistical analysis

A joinpoint regression analysis was used to assess yearly changes in the secular trends of cryoglobulinemia mortality rates [12]. The annual percentage changes (APCs, e.g., rate of 2000 − rate of 1999 × 100/rate of 1999) were computed for each segment of the trends, and the trends in the statistical tests were examined if they differed from zero. To assess whether there was a significant change in the proportionate mortality rate following introduction of the DAA agents, we calculated the 2014–2018/2009–2013 proportionate mortality rate ratio (PMR) and the 95% confidence intervals (95% CIs).

## Results

**Fig. 1:**
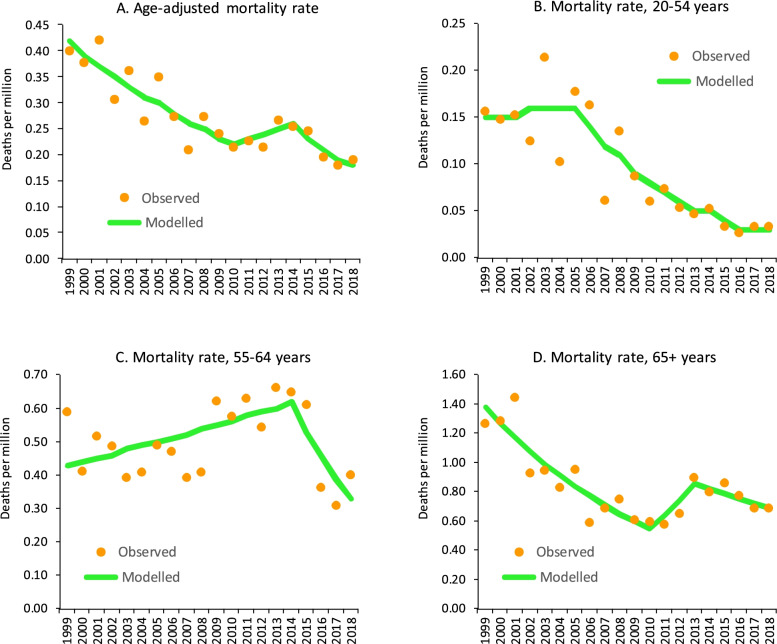
Cryoglobulinemia mortality rates in the USA, 1999–2018 (modeled rates were estimated according to joinpoint regression analysis)

We identified 1299 people aged ≥ 20 years who died between 1999 and 2018 and whose death certificates mentioned cryoglobulinemia. The number of deaths decreased from 80 in 1999 to 58 in 2018, and the age-adjusted cryoglobulinemia mortality rate (deaths per million) declined from 0.40 in 1999 to 0.19 in 2018. Three mortality trend segments for were identified for age-adjusted mortality rate: the first was related to the years 1999 to 2010 (APC: − 5.6%), the second to the years 2010 to 2014 (APC: 3.8%), and the third to the years 2014 to 2018 (APC: − 8.9%; Fig. 1A). The mortality rate for different age groups is illustrated in Fig. 1 B, C, and D, respectively. For decedents aged 55–64 years, the APC since 2014 was − 14.3% (*p* < 0.001) (Fig. 1C), and for decedents aged ≥ 65 years, the APC since 2014 was − 4.1% (*p* = 0.304) (Fig. 1D).

With regard to the demographic characteristics of decedents with cryoglobulinemia, a large decrease in the proportion of decedents aged 20–54 years was noted, from 30% (114/382) in 1999–2003 to 9% (27/310) in 2014–2018, whereas the proportion of decedents aged 65 years and above increased from 54% (207/382) in 1999–2003 to 60% (187/310) in 2014–2018 (Table 1).

**Table 1:** Number of deaths and mortality rates (deaths per million) with cryoglobulinemia in the USA, 1999–2018

	1999–2003			2004–2008			2009–2013			2014–2018			2014–2018/2009–2013	
	No.	(%)	Rate	No.	(%)	Rate	No.	(%)	Rate	No.	(%)	Rate	Rate ratio	(95% CI)
Total^a^	382	(100)	0.37	305	(100)	0.28	302	(100)	0.24	310	(100)	0.21	0.91	(0.76–1.05)
Sex														
Men	168	(44)	0.34	136	(45)	0.26	149	(49)	0.27	141	(45)	0.24	0.89	(0.68–1.10)
Women	214	(56)	0.41	169	(55)	0.30	153	(51)	0.26	169	(55)	0.27	1.05	(0.81–1.27)
Age (years)														
20–54	114	(30)	0.16	94	(31)	0.13	48	(16)	0.06	27	(9)	0.04	0.56	(0.29–0.82)
55–64	61	(16)	0.48	69	(23)	0.43	114	(38)	0.61	96	(31)	0.46	0.77	(0.55–0.97)
65+	207	(54)	1.17	142	(46)	0.76	140	(46)	0.67	187	(60)	0.76	1.13	(0.88–1.38)

^a^Rate of total was age-adjusted using age structure of US population of 2000 as reference

*95% CI* 95% confidence interval

The cryoglobulinemia mortality rates by sex and age in the four study periods are presented in Table 1. Two prominent reductions in mortality rates occurred in decedents aged 20–54 years: the first from 0.13 in 2004–2008 to 0.06 in 2009–2013 (50% decrease) and the second from 0.06 in 2009–2013 to 0.04 in 2014–2018 (44% decrease). By contrast, for decedents aged 55–64 years, an increase in mortality of 0.43 in 2004–2008 to 0.61 in 2009–2013 (40% increase) was noted, followed by a decrease in mortality from 0.61 in 2009–2013 to 0.46 in 2014–2018 (24% decrease). A minor increase in the mortality of those aged 65 years and over was noted: 0.67 in 2009–2013 to 0.76 in 2014–2018 (13% increase).

The proportion of decedents with cryoglobulinemia and HCV was 39% (118/302) in 2009–2013, but this decreased to 26% (81/310) in 2014–2018 with a PMR of 0.67 (95% CI 0.50–0.89) (Table 2). The PMR for each age group was lower than one, but it was not statistically significant. The proportion of decedents with cryoglobulinemia and systemic autoimmune diseases was 2.6% (8/302) in 2009–2013, but this increased to 4.2% (13/310) in 2014–2018, with a PMR of 1.58 (95% CI 0.66–3.82). SLE (*n* = 15) and Sjogren’s syndrome (n = 20) were the two main systemic autoimmune diseases associated with cryoglobulinemia.

**Table 2:** Numbers of people who died with mention of cryoglobulinemia and hepatitis C virus and those died with mention of cryoglobulinemia and systemic autoimmune disease in the USA, 1999–2018

	1999–2003		2004–2008		2009–2013		2014–2018		1999–2018		2014–2018/2009–2013	
	No.	%	No.	%	No.	%	No.	%	No.	%	PMR	95% CI
Cryoglobulinemia with hepatitis C virus												
Total	125	32.7	99	32.5	118	39.1	81	26.1	423	32.6	0.67	(0.50–0.89)
Sex												
Men	62	36.9	60	44.1	67	45.0	50	35.5	239	40.2	0.79	(0.55–1.14)
Women	63	29.4	39	23.1	51	33.3	31	18.3	184	26.1	0.55	(0.35–0.86)
Age (years)												
20–54	74	64.9	55	58.5	23	47.9	11	40.7	163	57.6	0.85	(0.41–1.74)
55–64	19	31.1	28	40.6	70	61.4	41	42.7	158	46.5	0.70	(0.47–1.02)
65+	32	15.5	16	11.3	25	17.9	29	15.5	102	15.1	0.87	(0.51–1.48)
Cryoglobulinemia with autoimmune disease (ICD-10 codes)												
Autoimmune disease (M30–M36)	16	4.2	13	4.3	8	2.6	13	4.2	50	3.8	1.58	(0.66–3.82)
Systemic lupus erythematosus (M32)	6	1.6	3	1.0	2	0.7	4	1.3	15	1.2	1.95	(0.36–10.64)
Sjogren’s syndrome (M350)	6	1.6	5	1.6	3	1.0	6	1.9	20	1.5	1.95	(0.49–7.79)
Other autoimmune disease	5	1.3	6	2.0	3	1.0	5	1.6	19	1.5	1.62	(0.39–6.79)
Sex												
Men	4	2.4	1	0.7	4	2.7	2	1.4	11	1.9	0.53	(0.10–2.88)
Women	12	5.6	12	7.1	4	2.6	11	6.5	39	5.5	2.49	(0.79–7.82)
Age (years)												
20–54	4	3.5	4	4.3	2	4.2	3	11.1	13	4.6	2.67	(0.45–15.96)
55–64	5	8.2	3	4.3	4	3.5	4	4.2	16	4.7	1.19	(0.30–4.75)
65+	7	3.4	6	4.2	2	1.4	6	3.2	21	3.1	2.25	(0.45–11.13)

*PMR* proportionate mortality ratio, *95% CI* 95% confidence interval, *ICD-10 codes* International Classification of Diseases, Tenth Revision

## Discussion

The findings of this national population-based study indicate that a persistent decline in the cryoglobulinemia mortality rates from 1999 to 2010, followed by a mild increase from 2010 to 2014, and then an abrupt decline from 2014 to 2018. The study has indicated high prevalence of HCV among baby boomers born 1945–1964 and resulted in the increase of HCV mortality since 2000 [13, 14]. The cryoglobulinemia mortality for middle-age adults aged 55–64 years increased since early 2000s (Fig. 1C) and for older adults aged ≥ 65 years increased since 2010 (Fig. 1D) corresponding to age-specific HCV mortality rates of baby boomers born 1950–1959 and 1945–194, respectively [14].

The abrupt decline in cryoglobulinemia mortality since 2014 among decedents aged 55–64 years and ≥ 65 years can be mainly attributed to the introduction of DAAs in 2014. However, the magnitude of the decline was more prominent among decedents aged 55–64 years (APC was − 14.3%) than among decedents aged ≥ 65 years (APC was − 14.3%), which might be due to different access to DAA among two sub-cohort baby boomers [14]. The proportion of patients with cryoglobulinemia and HCV reported in a French study was 63% in 2011, 60% in 2015, and 33% in 2018 [10]. In this study, the proportion of decedents was 37% in 2011, 30% in 2015, and 26% in 2018.

With regard to the autoimmune diseases, the proportion of decedents with cryoglobulinemia who had systemic autoimmune diseases increased from 17% in 2011 to 36% in 2018 in the French study [10]. Similar increases were observed in this study: the proportion was 2.6% in 2009–2013 and increased to 4.2% in 2014–2018.

One strength of the present study is that it used national population-based mortality data to examine the long-term cryoglobulinemia mortality trends and changes in association with HCV. To our knowledge, this study included a greater number of cryoglobulinemia cases and a larger observational year span than previous studies. However, several limitations should be noted when interpreting the findings of this study. First, some physicians may underreport either cryoglobulinemia, HCV, or systemic autoimmune diseases on death certificates. The comparison between proportions represented in this study and those in the French study suggest that the extent of underreporting may differ depending on the disease reported. For example, a study of death certificates in Connecticut revealed that among deaths for which hepatitis B, hepatitis C, and alcoholic liver disease were identified during the medical record review, the specified ICD codes were only recorded on 8.6%, 45.4%, and 36.5%, of the death certificates, respectively [15]. However, our primary aim was to examine mortality trends, and it is unlikely that there would have been substantial changes in the underreporting behaviors of certifiers during the study period.

Second, it was not possible to further analyze mortality trends by cryoglobulinemia type, because the ICD-10 code for cryoglobulinemia does not further classify types. Third, as no information on clinical laboratory data were available from mortality data, it was not possible to stratify the decedents into different clinical profiles. Fourth, because only a small number of deaths had occurred, we did not further analyze data by race and place of residence. Fifth, information relating to whether the patients received DAA treatment was not available. These limitations show that further studies linking health care services data with mortality data are required.

## Conclusion

In conclusion, a decline in cryoglobulinemia mortality occurred in the USA that began in the early 2000s, leveled off between 2010 and 2014, and then drastically declined between 2014 and 2018. The changes in mortality trends patterns are considered to be mainly attributed to the aging and dying of a “baby boomer” cohort who had a high prevalence of HCV and to the introduction of DAAs in 2014. Rheumatologists should screen patients with cryoglobulinemia if they had HCV infection and urge patients with cryoglobulinemia and HCV receiving DAA treatment to reduce the risk of death.

## Data Availability

The data are available upon the request to the corresponding author.

## Abbreviations

APC: Annual percent change
ASDR: Age-standardized death rate
DAA: Direct-acting antivirals
HCV: Hepatitis C virus
ICD-10: International Classification of Diseases Tenth Revision
MCOD: Multiple cause of death
NCHS: National Center for Health Statistics
PMR: Proportionate mortality ratio
SLE: Systemic lupus erythematosus
95% CI: 95% confidence intervals
